# *In vivo*, *in vitro* and *in silico*: an open space for the development of microbe‐based applications of synthetic biology

**DOI:** 10.1111/1751-7915.13937

**Published:** 2021-09-27

**Authors:** Antoine Danchin

**Affiliations:** ^1^ Kodikos Labs Institut Cochin 24 rue du Faubourg Saint‐Jacques Paris 75014 France

## Abstract

Living systems are studied using three complementary approaches: living cells, cell‐free systems and computer‐mediated modelling. Progresses in understanding, allowing researchers to create novel chassis and industrial processes rest on a cycle that combines *in vivo*, *in vitro* and *in silico* studies. This design–build–test–learn iteration loop cycle between experiments and analyses combines together physiology, genetics, biochemistry and bioinformatics in a way that keeps going forward. Because computer‐aided approaches are not directly constrained by the material nature of the entities of interest, we illustrate here how this virtuous cycle allows researchers to explore chemistry which is foreign to that present in extant life, from whole chassis to novel metabolic cycles. Particular emphasis is placed on the importance of evolution.

## Graphical abstract

The iteration cycle: design–build–test–learn, between experiments and analyses combining physiology, genetics, biochemistry and bioinformatics in an ever more advanced way, generates the most interesting future developments in microbial synthetic biology of industrial interest.

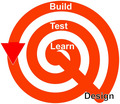

## Introduction: complementary avenues for the exploration of life

It took a long time for science to understand that there was no difference between ‘organic’ and ‘inorganic’ matter. The nature of the chemical compounds of life had no specificity other than, perhaps, the fact that they were based on the chemistry of carbon. Still, the status of biology is somewhat different from that of standard chemistry, for living organisms are deeply connected with a surprising quality of ‘animation’, which at first sight seems alien to the laws of physics. As a consequence of this observation, studies of living organisms were separated into *in vivo* (using living cells and organisms as the basic entities of interest) and *in vitro* (using parts of living entities combined together in test tubes) studies. More recently, with the enormous development of computing facilities, it has become possible to replace experimental studies using authentic material entities with studies of the living using computers. Although little developed before the advent of DNA sequencing techniques, this change in the study of life was greatly stimulated when it became apparent that knowledge of genomic sequences would rapidly become ubiquitous. The need to develop this endeavour was highlighted for the first time at a meeting of the European Biotechnology Action Programme in Tutzing, Germany, in 1989: ‘*From the information technologies perspective, we are faced with the need to create databases where we will recognize relevant features in response to learning processes. The creation of descriptors, the genesis of exploratory hypotheses will add to the traditional methods of biology,* in vivo *and* in vitro*, experiments* in silico’. Although this emphasis is already three decades old, it can be safely anticipated that *in silico* approaches will gain in importance in the coming decades, especially for developments in microbial biotechnology. In what follows, we briefly summarize the state of *in vivo* microbial studies, focusing on the forthcoming development of novel artificial chassis, then focus on current trends in cell‐free syntheses and conclude with progress in *in silico* research. One of the conclusions of this study is that to understand the functions responsible for the apparent animation of living systems, the activities that dissipate energy in unanticipated ways should be given priority in the coming years.

## A future for *in vivo* microbial studies

Microbial biotechnology exploits a substantial collection of microbes widely used to generate products/processes of interest to industry. Industrial applications go hand in hand with understanding the puzzles of their environmental niches and metabolism. We can anticipate that, using these same organisms, metabolic engineering will continue to evolve over the next few decades, now benefiting from rapid advances in synthetic biology (SynBio), with a likely emphasis on the use of large‐scale DNA synthesis (Panke, 2020). This topic has been the subject of many studies [see, e.g. Calero and Nikel (2019)], and we do not explore it further here, except for a brief review of recent uses showing how novel living chassis will gradually gain impact in the near future. Following this pattern, we describe in this section how the emergence of novel microbial chassis is about to revolutionize microbial engineering.

### Brief landscape of the use of genetically modified natural microbial chassis

Until recently, the majority of microbial biotechnology advances that did not rest on microbes with idiosyncratic properties – typically, specific synthesis of metabolites such as vitamin B12 (Balabanova *et al*., 2021) – used a variety of *Escherichia coli* strains as the basis for elaborate metabolic engineering. This is not discontinued: sophisticated SynBio designs are already implemented in this organism, as seen, for example, in the use of optogenetics to control expression of the plant lycopene biosynthesis pathway in this organism (Raghavan *et al*., 2020). Such innovative leads could soon become routine. The ubiquitous role of *E. coli* as a chassis was not unexpected due to the considerable accumulation of knowledge gained for this species. Relevant access to accurate sequence annotation is key for productive industrial developments. Alas, errors keep percolating in automatic genome annotation procedures that are in effect essentially based on the still imperfect knowledge of model organisms (Bell and Lord, 2017). Yet, complete, up‐to‐date annotation of the *E. coli* genome sequence is unfortunately not properly maintained on a perennial basis. The best public annotation of the sequence at this time is perhaps the one developed using orthologue‐based annotation propagation (Paley *et al*., 2021), but it is not adapted to the subtle requirements of industrial demands. This drawback implies that the use of chassis other than *E. coli* requires even more tedious manual annotation to account for the inevitable idiosyncrasies uncovered in the course of engineering [see the importance of ‘kludges’, by definition unexpected, in (Danchin, 2021)].

SynBio studies have long aimed at getting rid of seemingly superfluous gene‐encoded functions, creating universal minimal chassis in which biotechnology‐relevant pathways can be introduced. In line with this objective, the Mollicutes clade was perceived as an interesting paradigm because these organisms had already evolved streamlined genomes (Piñero‐Lambea *et al*., 2020). Mollicutes have been and will still be used to understand the minimal constraints of cell survival and multiplication (Garcia‐Morales *et al*., 2020). However, because these organisms have a highly reduced genome and minimal macromolecule synthesizing machineries, they are intrinsically fragile and limited in their metabolic power, and thus will hardly be convenient for widespread industrial developments. In contrast, a large collection of organisms that thrive in more or less extreme environments are likely to take the limelight in the near future (Liu and Deutschbauer, 2018). Here is a brief list of some relevant candidates [see (Calero and Nikel, 2019) for a detailed account of an exhaustive list of non‐traditional industrial microbes.]

The Pseudomonas genus is well suited to metabolize aromatic compounds and harbour novel metabolic pathways (Bitzenhofer *et al*., 2021). The genome of *Pseudomonas putida* has already been streamlined and shown to allow improved heterologous gene expression (Lieder *et al*., 2015). A variety of tools are available for this organism, which now becomes a widespread platform for SynBio constructs (Martin‐Pascual *et al*., 2021). For example, even though this organism has its metabolism poised to develop in the presence of dioxygen, strain KT2440 has been modified to multiply in anaerobic conditions (Kampers *et al*., 2021). A companion, *Comamonas testosteroni,* a species belonging to a related clade, has become a promising chassis for bioremediation due to its natural pollutant‐degrading capacity (Aksu *et al*., 2021).

In the actinobacterial clade, *Corynebacterium glutamicum* has been used for decades as a major industrial source of vitamins, amino acids, carbohydrates and a variety of other metabolites. It is now the subject of a fair number of improvements to accommodate novel metabolic pathways and SynBio constructs (Chen *et al*., 2021a; Wang *et al*., 2021; Wolf *et al*., 2021). With many industrial facilities already using this organism, it seems likely that further improvements will accumulate rapidly. In the same way, but in the Firmicutes clade, *Bacillus subtilis* has also been much discussed as an important industrial chassis (Liu *et al*., 2020; Xiang *et al*., 2020). Besides applications similar to those discussed previously with other organisms, as well as often ignored roles, such as that in heavy industry where it is used for improving concrete ageing *via* creating self‐healing properties, for example (Nielsen *et al*., 2020), the fact that Bacilli generate extremely resistant spores is likely to generate original methods for new types of applications. Using a 3D printer, Christopher Voigt and his colleagues printed *B. subtilis* spores within an agarose scaffold, showing that they germinated on its exterior surface, including spontaneously in cracks. The agarose‐spores material sustained desiccation and returned to life after rehydration. When containing spores of *B. subtilis* engineered to produce antibiotics, the material could be used to kill *Staphylococcus aureus*, a pathogen causing a variety of infections (González *et al*., 2020). This family of advances are so interesting that they have even been used for 3D printing of a mixture of recombinant *E. coli‐*expressing metallothionein/gold nanocomposites used as agitating paddles for the catalytic reduction of 4‐nitrophenol (Long *et al*., 2021).

Still in the domain of bacteria, Cyanobacteria, being able to fix carbon dioxide, have a distinctive status, especially under conditions when the steady increase in carbon dioxide in the atmosphere of our planet is a matter of concern. The species of this phylum are likely to become a preferred chassis for biotechnological developments in the near future (Wang *et al*., 2020). However, they display specific structural features that make them difficult to manipulate. Their metabolism, resting on gasses and reactive metabolites, must be compartmentalized (de Lorenzo *et al*., 2015; Flechsler *et al*., 2021). This means that future progress with these organisms is likely to require considerable effort to account for the existence of compartments before they are suitable for versatile metabolic engineering. Nevertheless, the proliferation of research facilities based on this family of chassis is already showing us how these bacteria will soon be commonly used (Yang *et al*., 2020; Cui *et al*., 2021). Other photosynthetic bacteria have also been proposed as new chassis, especially for water treatment. Some non‐sulphur bacteria, the oldest of the photomicroorganisms, not only treat different types of wastewater but are also non‐toxic bioresources, containing many value‐added products. These bioresources can be used as raw materials in the agricultural, food and medical industries (Lu *et al*., 2021).

Finally, besides a few applications using Archaea [see, e.g. Aldridge *et al*. (2021)], the Eukarya domain of life is also a common source for microbial biotechnology. In this context, the model *Saccharomyces cerevisiae* plays a role similar to that of *E. coli*, but other yeasts are also widely used, such as *Yarrowia lipolytica*, for lipid‐related applications (Park and Nicaud, 2020). The lines of research we have just outlined with Bacteria will also be developed with these organisms, including after their genomes have been reworked to facilitate genetic manipulation (Schindler, 2020). This is already seen in the high‐level original developments such as those listed in Voigt (2020). These examples demonstrate that we are on the verge of an enormous number of applications based on SynBio *in vivo* metabolic engineering and material improvement of extant microbes, notwithstanding the variation associated with inevitable evolution of all living organisms (Wassenaar and Zimmermann, 2020).

### Alien chassis and xenobiotics

Most contemporary advances in SynBio are variations on a theme based on reconstructed metabolic pathways derived from natural sources, and artificial constructs deployed in a variety of natural chassis. Conclusive innovation, however, will really come to life when heterologous implementation of known pathways will be replaced by counterparts that have not evolved in extant organisms, as well as chassis modified to be distinct from standard living cells. A considerable amount of work is being done in this direction. Here, we describe some of the corresponding endeavours, while recognizing that, for the time being, examples of modification of the core chemistry of extant chassis remain rare. Perhaps the best illustration of promising work is the creation of *Escherichia chlori*, an avatar of *E. coli* where thymine has been entirely replaced by 5‐chlorouracil in DNA (Marlière *et al*., 2011). However, there is only little data available about the stability of the construct. We should expect to understand before long the functions that have been altered in this organism, allowing it to grow on 5‐chlorouracil while being inhibited by thymine. This entails understanding how these chassis will behave in the long run, but we can be confident that there is some hope for stability as demonstrated in stable evolution after 100 days of an *E. coli* strain after continuous co‐culture with mammalian cells (Kunjapur *et al*., 2021).

A large number of avenues are being explored to further modify the nature of the genetic material of cells based on xenonucleic acids (Fiers *et al*., 2016). While there is continuous progress in the domain (Chaput and Herdewijn, 2019), the emergence of autonomous novel chassis amenable to industrial applications seems unlikely for the next decade or so. In the meantime, less dramatic changes continue to be designed. Since the beginnings of SynBio research, researchers attempted to reassign codons (Robertson *et al*., 2021), often to non‐natural proteinogenic amino acids, as for example illustrated early on (Döring and Marlière, 1998). The most efficient approaches attempt to device orthogonal constructs, for the time being devoted to a limited fraction of the translation machinery (Schmied *et al*., 2018). Recent attempts have created an artificial genetic code of 68 codons that could allow the incorporation of four non‐canonical amino acids into synthetic proteins (Dunkelmann *et al*., 2021). In other advances, the synthesis of proteins containing d‐amino acids, β‐amino acids, phosphorylated amino acids as well as long‐chain and cyclic amino acids in which the nucleophilic amino group is not in the α‐position has been achieved using modified peptidyl transferase regions in the 23 ribosomal RNA. Dipeptides and dipeptidomimetics of varying utility were also obtained (Hecht, 2021). Because this is sophisticated work – relevant cells must maintain both the standard machinery and an orthogonal one – industry‐relevant progress has been slow. With an increasing number of laboratories involved in these attempts, the number of successes is expected to increase rapidly. However, again, much of the corresponding works are more proofs of concept than ready to become, biotechnology‐adapted synthetic microbes growing in industrial fermenters. More limited approaches have already been used to produce synthetic variants of proteins, for example, using analogues of amino acids such as selenomethionine for bioconjugations (Flood *et al*., 2021) or variants of norleucine, known to replace methionine in proteins in *E. coli* for a very long time (Moroder and Budisa, 2010; van Eldijk and van Hest, 2018). Nevertheless, a creation somewhat similar in its concept to that of *E. chlori* was based on growth of cells on tryptophan analogues. In the earliest experiments, full replacement of tryptophan residues by an analogue could not yet be achieved (Bacher and Ellington, 2001). Later on, complete replacement was successful in a *B. subtilis* mutant that swapped all tryptophan residues for 4‐fluorotryptophan after serial selection and mutagenesis (Yu *et al*., 2014).

Yet another highly focused advance, isobiology, has been proposed. It is based on the observation that biological processes differentiate the isotopes of atoms used to form cells. This line of experiments involves a very small modification of the chassis. It is, therefore, likely to have minimal consequences. The most important effect is expected when the standard cell composition replaces hydrogen by deuterium, in particular when heavy water (D_2_O) replaces standard water (H_2_O). Notwithstanding its financial cost, this procedure limits the consequences of changes while allowing the fine properties of the cell chassis to be explored (Danchin, 2020). This type of effort is likely to be important for understanding functional phase transitions in cells (Choi *et al*., 2020), a concept that emerged in parallel with the onset of SynBio, and which is likely to be crucial in the construction of novel chassis as it defines previously neglected membrane‐less compartments (Gomes and Shorter, 2019). As an illustration of future progress, specific isotope‐sensitive microbial models could be used for the development of biotechnological processes, based on the behaviour of thermophilic Archaea (Nishizawa *et al*., 2016). This would be motivated, for example, by the fact that the use of carbon, nitrogen and oxygen isotopes is already well accepted – despite the cost of the experiments – as safe substitutes for tracer experiments (Davies, 2020). This opens up a niche for the industrial microbial production of amino acids and nucleotides (Nicolás Carcelén *et al*., 2017).

### Adaptive evolution

Life is a dynamic process, not only during the lifetime of an organism but also in the long term, as it keeps generating offspring. Evolution is an essential feature of life. Therefore, many SynBio projects have enabled constructs established in well‐identified laboratory strains to evolve under various conditions [see, e.g. Li *et al*. (2021)]. Adaptive evolution has even been successfully developed for co‐evolving communities of organisms (Konstantinidis *et al*., 2021). In most cases, the operation has developed in large volume vessels, controlled as chemostats are. Among promising approaches, a variation on chemostats driven by growth medium dilution found that, in a continuous culture, growth chambers were invaded by mutants that escape dilution by attaching to the surface of the vessel. As expected, this trait is positively selected. It generates persistent subpopulations whose size and growth rate are uncontrollable, preventing the use of standard detection methods such as turbidity measurements to monitor growth. To overcome this limitation, a device with two growth chambers subjected to brief periodic alkaline sterilization phases has been designed (de Crécy‐Lagard *et al*., 2001; de Crécy *et al*., 2007). Yet, because these vessels are at least a few millilitres in volume, this setting precludes carrying out large‐scale experiments with many vessels working in parallel. This drawback triggered the development of continuous culture systems based on open reactors managing microfluidic droplets [see (Ito *et al*., 2016) and the special issue on recent advances of molecular machines and molecular robots edited by Takinoue and Kawano, which describes advances in the domain, mainly in Japan (Takinoue and Kawano, 2020)]. The future of laboratory‐set evolution is now likely to rest on nanolitre reactors such as those already used for production of important metabolites (Xu *et al*., 2020), allowing *in vivo* directed evolution (Femmer *et al*., 2020). This is at the cost of the evolving population's size, however, so that it is likely that a significant family of evolution devices will keep using sizeable vessels in the future.

### The design of alien metabolism

**Fig. 1:**
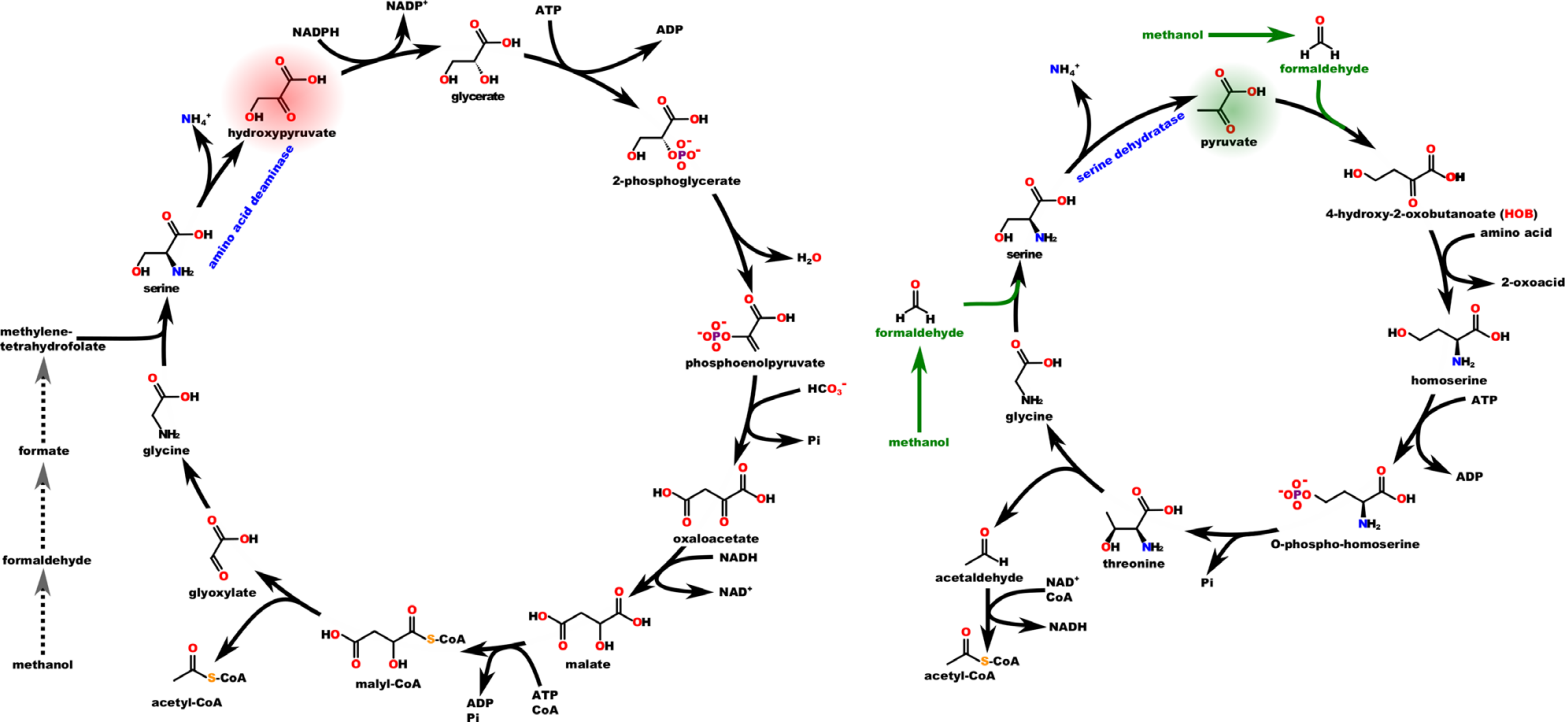
Natural methanol assimilation compared with methanol assimilation in a HOB‐dependent cycle. The usual input of methanol/formate into carbon metabolism (left) is compared (right) with the novel HOB‐dependent cycle described in He *et al*. (2020). Note that hydroxypyruvate is potentially toxic.

Intermediary metabolism is an intrinsic component of all live chassis. In most cases, researchers have attempted to streamline their pathways of interest in existing organisms with a small series of efforts to completely replace essential pathways with artificial ones. Still, extant pathways remain important: *E*. *coli* can be reprogrammed to fix carbon dioxide by simple modifications in its canonic carbon metabolism pathways (Satanowski *et al*., 2020). However, an entirely novel pathway for carbon fixation could be implemented in this organism. The standard core one‐carbon metabolism has been reprogrammed with the design of an alternative cyclic pathway that substitutes 4‐hydroxy‐2‐oxobutanoic acid (HOB), a compound absent from canonical metabolism – except perhaps as an accidental consequence of aminotransferases’ promiscuity (Walther *et al*., 2018) –, for the amino acids serine and glycine as omnipresent one‐carbon donors. This pathway is based on two novel reactions, transamination of l‐homoserine and transfer of a one‐carbon unit from HOB to tetrahydrofolate, releasing pyruvate. Canonic reactions regenerate l‐homoserine from pyruvate by carboxylation and subsequent reduction. In this way, any one‐carbon moiety made available for anabolic reactions may originate from CO_2_. The HOB‐dependent pathway was established in an *E. coli* auxotroph selected for prototrophy after enrichment in a long‐term cultivation system (Marlière *et al*., 2011). Monitoring its stability will be interesting, as l‐homoserine, an analogue of authentic proteinogenic amino acids, interferes with translation (Jakubowski, 1997). Living organisms have found solutions to circumvent this obstacle, for example, via protection/deprotection systems (Danchin and Sekowska, 2015), and we should expect that, if the pathway’s industrial role requires to raise its production level, such solutions will emerge before long in laboratory‐set evolution experiments. Indeed, a cognate cycle that uses a homoserine phosphorylation step was successfully designed to use methanol to feed general SynBio‐derived bioproduction processes, and it likely to be improved in the near future [(He *et al*., 2020) and Fig. 1].

While it remains essential for SynBio developments to design integrated living units, the very fact that living cells are constantly evolving makes it crucial to bring out the symmetry of synthesis and analysis – breaking down cells into their components – both to understand the details of life processes and to implement industry‐friendly activities that are more stable over time than inventions with life. This involves exploring the principles of synthetic life *in vitro*, bearing in mind that we must both master the choice and availability of relevant components and advance the conceptual understanding of all the relevant processes that take place in living cells in order to make them work *in vitro*.

## *In* *vitro* syntheses and evolution

Molecular biology is a combination of genetics and biochemistry (https://plato.stanford.edu/entries/molecular‐biology/). Until these fields of biology were merged, the study of life was divided between the exploration of organisms *in vivo* – alive – and the chemical analysis of some of their components once synthesized in test tubes, *in vitro*. However, what was successful *in vivo* did not overlap much with what was discovered *in vitro*. The apparent ‘animation’ of cells and organisms remained a mystery. As in the early XIX^th^ century, when chemicals were divided between organic and inorganic matter, some biologists believed – some still believe – that macromolecules, in particular, have mysterious properties, specific to life. The actual, purely chemical synthesis of macromolecules was, therefore, a prerequisite for the exploration of life, accepted as belonging to the realm of physics and chemistry. This feat was achieved with small proteins such as insulin. Today, the chemical synthesis of proteins is a difficult challenge that remains relevant, not in response to philosophical debates, but as an industrial target (Hui *et al*., 2021): high‐level accuracy in the protein sequence of amino acids appears to be easier to control *in vitro* than *in vivo* (Karas *et al*., 2021). The same situation prevailed with nucleic acids (Caruthers, 2013), with the same consequences in terms of industrial developments: For example, gene and vaccine syntheses require a very high precision, with accessible controls at each stage, a requirement which remains still difficult to achieve with *in vivo* constructs. While life seems to have evolved a way to keep error rate in many processes to some minimum (Bradley *et al*., 2019; Mordret *et al*., 2019), the trade‐off between speed and accuracy in biosyntheses has been adopted by natural selection as a means to input resilience in the build‐up of living entities (Danchin *et al*., 2011), making a significant level of errors an inevitable consequence (and cause) of evolution (Médigue *et al*., 1991; Piñeros and Tlusty, 2020; Jones and Uphoff, 2021).

### Cell‐free protein synthesis, a half‐century ongoing story

Once it was recognized that there was no magic involved in enzymatic activity, it was widely – but not always – accepted that enzyme‐based synthesis of molecules *in vitro* could be accepted as yet another artificial means of constructing macromolecules to complement purely chemical syntheses. In a first step – in an experiment with impact similar to that of the synthesis of urea by Woehler, as it established that there was no animation principle in RNA synthesis –, Sol Spiegelman used the replicase of RNA bacteriophage Qβ to synthesize phage RNA in the test tube. The ‘little monster’, thus, produced was physically and chemically indistinguishable from the original virus RNA. Indeed, to establish that the test‐tube RNA was infective, Spiegelman injected it in a solution of all four nucleoside triphosphates and Qβ replicase, waiting for the RNA to replicate. He then incubated it with live bacteria, and demonstrated that the RNA led to generation of infective viral particles (Haruna and Spiegelman, 1965). In another family of *in vitro* experiments, RNA synthesis of more or less random sequence was successful with an enzyme that did not use nucleic acids as templates, polynucleotide phosphorylase (Grunberg‐Manago, 1963). Much of the discovery of the genetic code table used synthetic RNA made with this enzyme, further blurring the line between chemistry and biology.

With synthesis of RNA and proteins available *in vitro*, the goal was to shift experiments to the upper level of biosyntheses and set up the multi‐level process of translation in the test tube. Mimicking macromolecule biosynthesis as it unfolds *in vivo* was a true challenge. To go further in the direction of authentic protein synthesis, it was important to demonstrate translation of a monocistronic message. This was established early on (Clark *et al*., 1965). This difficult undertaking, which began in the early 1960s, is still under development as more and more components are integrated into the synthetic machinery of test tube. We will gradually gain a better understanding of the reasons for the difference between these processes when they take place *in vivo* and *in vitro*. With synthetic RNAs, the importance of the pH and the role of magnesium (Jiang *et al*., 2021), polyamines (Fukuma and Cohen, 1975) and potassium (Fritz *et al*., 2018) were identified as crucial for success (Hammerling *et al*., 2020). Translation with ribosome preparations, endowed of progressively increasing specificity and accuracy, was much improved after the discovery of the need to start and stop translation, using initiation factors (Clark and Marcker, 1966) and subsequently other factors such as the ribosome release factor (Kung *et al*., 1977). A further step was gained when DNA, much more stable than RNA, could be used as a template for *in vitro* gene expression, allowing protein synthesis *via* coupling transcription with translation in the test tube (Austin and McGeoch, 1973; Isturiz and Wolf, 1975).

Improved ‘cell‐free’ RNA and protein synthesis systems based on bacterial and eukaryotic extracts then became common, with cloned or synthetic genes as DNA templates (Billerbeck *et al*., 2013; Caschera and Noireaux, 2014; Li *et al*., 2014). The knowledge developed in the early decades of transcription–translation coupling in the test tube has been used to design a prototype flow microreactor for synthetic biology *in vitro* providing solutions for industrial biosyntheses (Boehm *et al*., 2013). Reconstituted cell‐free protein synthesis systems such as the protein synthesis using recombinant elements (PURE) system allowed synthesis of a wealth of recombinant proteins. This family of cell‐free systems keeps being improved (Grasemann *et al*., 2021). Yet, despite continuous progresses (Zhang *et al*., 2021b), sometimes for industrial purposes (at least for the synthesis of proteins used by research laboratories), the speed and accuracy of the translation process remained significantly below what happens *in vivo*.

Several biological functions were identified as critical for improving the outcome of cell‐free syntheses. For example, proper protein folding was critical. In contrast to the widely accepted, but wrong, Anfinsen’s postulate that assumes that the whole folding potential is present in the protein primary sequence, it is now established that most unfolded proteins cannot spontaneously fold properly (To *et al*., 2021). As a first step, the ribosome itself is important as a folding device during translation, but the trigger factor (Piette *et al*., 2011; Koubek *et al*., 2021) and other molecular chaperones (Yang *et al*., 2019) have often a decisive role. Many are dissipating energy (Boel *et al*., 2019; Mallory *et al*., 2019), which impacts the energy balance of the procedure, a feature rarely discussed. Furthermore, it appeared progressively that the role of water and molecular crowding were critical to ensure fidelity of translation [see, e.g. Garenne *et al*. (2020)]. The importance of these functions is not yet fully understood and it is likely that their role will impact future developments of cell‐free syntheses. Their role is to discriminate between classes of entities (young and aged objects and also spatial positions or alternative structures, for example). This is illustrated, for example, by innate immune sensors such as RIG‐I, which sensitively detect and respond to viral RNAs that enter the cytoplasm, while remaining unresponsive to the abundance of structurally similar RNAs that are the products of host metabolism (Ren *et al*., 2019). Similarly, the SecA ATPase, which binds its substrates post‐translationally, scans the ribosomal tunnel for potential substrates to secrete the correct ones (Knüpffer *et al*., 2019). The information for discrimination is managed by the energy dissipation reflected by the ATP dependence of the enzyme (Boel *et al*., 2019). In terms of SynBio developments, consideration of these agents, particularly ATP‐dependent proteases such as protease Lon, will have a considerable impact on the production of canonical proteins (Tzeng *et al*., 2021).

Yet another idiosyncratic feature of proteins has also been identified as having a likely impact on cell‐free protein biosynthesis. While proteinogenic residues are universally considered to be ‘amino acids’, proline is a secondary cyclic amine (formerly, called ‘imino acid’). This characteristic requires the translation machinery to take this chemical singularity into account. Fast and accurate translation of proline residues requires a specific variant of a post‐translationally modified translation elongation factor, EF‐P, or other counterparts depending on the organism (Hummels and Kearns, 2020). Yet, the explicit use of EF‐P has seldom been explored as an explicit partner in cell‐free protein synthesis (Li *et al*., 2017; Hammerling *et al*., 2020). Sequences of three consecutive prolines can fold into polyproline helices, structures that join alpha helices and beta folds as architectural motifs in protein configuration. Triproline helices are involved in protein–protein signalling interactions. Management of elongation factor EF‐P in cell‐free synthesis of proteins with series of proline residues should improve the outcome of a protein production line. This is not a minor point, as, for example, more than 4% of the human proteome contains series of three or more prolines (Morgan and Rubenstein, 2013). Furthermore, since EF‐P binds to the 9 nt D‐loop found in tRNAPro isoacceptors, and not to proline itself (Katoh *et al*., 2016), the use of this factor can be expected to play a key role in the precision of cell‐free translation engineering, a feature essential for SynBio developments. After half a century of hard work, the various obstacles to the development of *in vitro* synthesis continue to be improved, with the emphasis on speed (Burrington *et al*., 2021). Much remains to be understood to allow the industrial process of translation to be stable in time, with proper maintenance activities in particular. We may expect that exploring these weak points will dominate research in the domain in the near future. This is more important because, during the spontaneous process of protein ageing – which is protein specific and spanning a considerable time frame, from minutes to centuries (Zhang *et al*., 2020) –, proteins tend to aggregate after subtle conformation changes in the absence of the energy requirements that allow a subset of NTP‐dependent activities to refold proteins in their proper shape, dissolving aggregates (Dewachter *et al*., 2021).

**Fig. 2:**
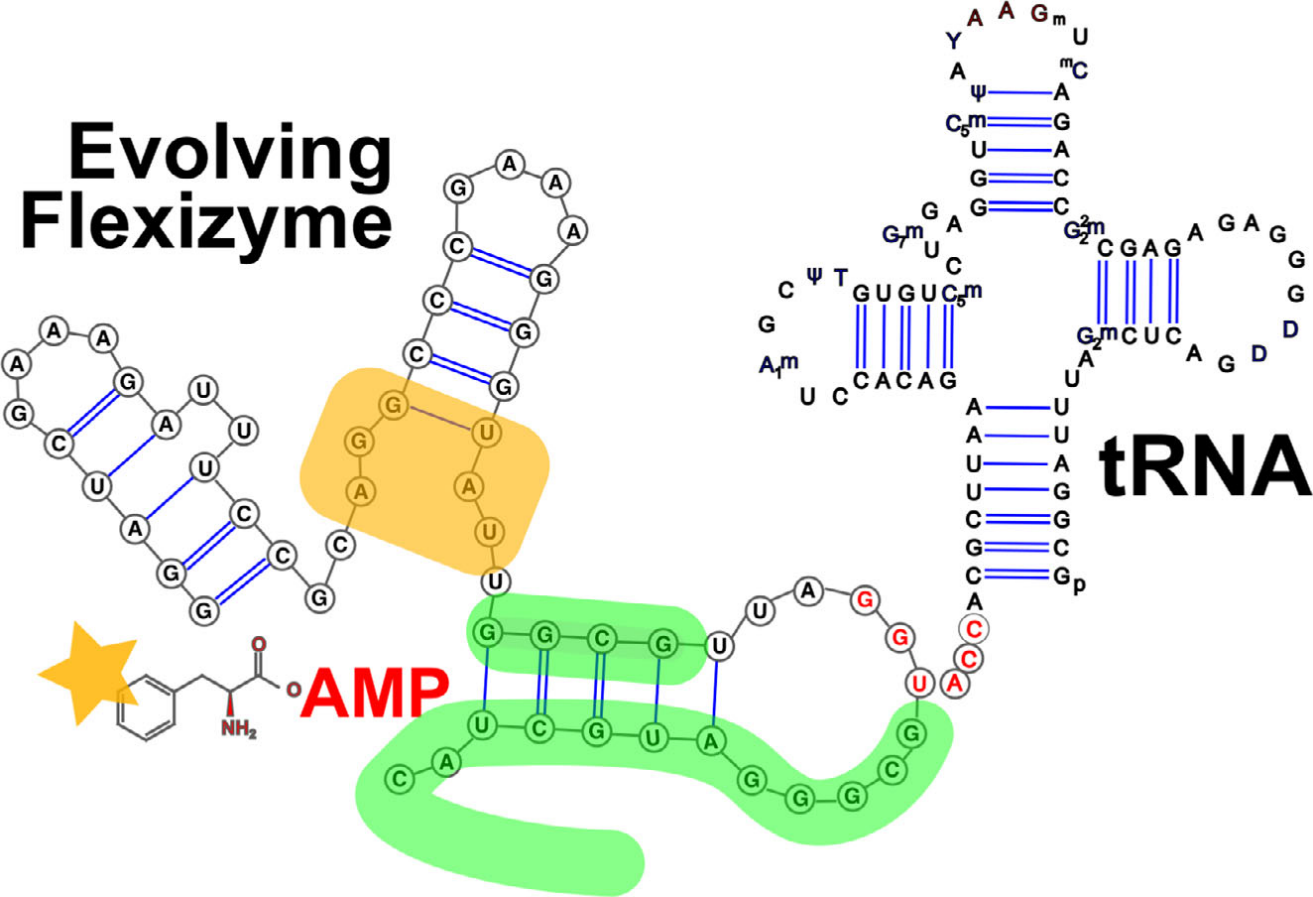
Flexizyme RNAs as substitutes for aminoacyl‐tRNA ligases [from Ohuchi *et al*. (2007)]. Starting from an aptamer that binds phenylalanyl‐AMP, synthetic variants (random sequences in the region highlighted in green) are constructed and submitted first for selection for binding to the 3’‐CCA end of phenylalanine tRNA, then to a further selection after variants (region highlighted in orange) are proposed for the binding of analogues of phenylalanyl‐AMP (orange star).

In addition to using *in vitro* protein synthesis as a means of identifying all the key partners of the *in vivo* process, cell‐free protein synthesis also allows proteins to be synthesized with a variety of non‐proteinogenic amino acid analogues. Worth highlighting, the ‘flexizyme’ system based on ribozymes that are able to load a large number of chemical substrates onto tRNAs [(Passioura and Suga, 2014) and Fig. 2] eliminates the need to modify aminoacyl‐tRNA synthetases prior to synthesis. We can expect that this family of cell‐free approaches will make a considerable part of next‐generation SynBio developments, in particular for the synthesis of polymers of industrial interest. This will enable the synthesis of polymers involving structurally disparate non‐α‐amino acids that bear little resemblance to canonical proteinogenic amino acids. Examples include long‐chain carboxylic acids, substituted benzoic acids, 1,3‐dicarbonyls, peptides and helical aromatic oligomer‐peptide hybrids. For example, substituted benzoic acids are the precursors of aramids, a class of aromatic polyamides with a rich progeny. Similarly, long‐chain amino acids are monomeric precursors of nylon (Tharp *et al*., 2021).

These novel trends will benefit both from development of RNA SynBio and from streamlined cell‐free systems amenable to scaling up.

As their name suggests, cells are compartments, specifically delimited by membranes built around lipid bilayers. Early on, as it became apparent that DNA‐driven *in vitro* protein synthesis was often difficult to achieve, some researchers resorted to the spontaneous ejection of chromosome‐less vesicles by some cells as an alternative to a purely *in vitro* system (Bose *et al*., 2020). These vesicles, ‘mini‐cells’, often kept plasmids in their cytoplasm, allowing protein expression from artificial constructs (Grindley *et al*., 1977; Miller *et al*., 1977; Bose *et al*., 2020). Similarly, in animal cell‐free systems, exosomes or cell‐derived small extracellular vesicles have been used in therapeutic intervention (Pedrioli *et al*., 2021). Lipid vesicles have also been used for some time and they are now used for glycosylation of proteins in cell‐free systems (Hershewe *et al*., 2021). Among the various hurdles still remaining to resolve are the synthesis of the lipid bilayer of the cell‐free systems membranes (Bhattacharya *et al*., 2021) as well as routine synthesis of active membrane proteins. Novel transcription–translation systems are now able to express integral membrane proteins. This allowed researchers to highlight the importance of lipid–protein interaction in maintaining structural–functional integrity of ion channels (Vaish *et al*., 2018). Membrane‐augmented cell‐free systems have been used in a wide array of cellular processes from primary and secondary metabolite synthesis to electron transport and one‐carbon metabolism (Kruyer *et al*., 2021).

Following on from Spiegelman’s early experiments, an important step in understanding the boundary between *in vivo* and *in vitro* syntheses, the synthesis of a fully active virus has been achieved (Rustad *et al*., 2017). While viruses cannot be considered alive – they need a live cell to multiply – the very fact that they can be made *in vitro* blurs further the boundary between chemistry and biology. Still, the cell‐free systems used to achieve this milestone are extracted from living cells, and are far from sufficient to be able to duplicate spontaneously. Nevertheless, this achievement paves the road to identify the biological functions that are responsible for the signature ‘animation’ of live cells (Danchin, 2021). However, much work remains to be developed to implement them knowingly in cell‐free systems. We can expect that this will be understood in a not too far away future, in particular if we understand better the role of apparently expletive energy‐dissipating functions, such as those involved in the control of folding and aggregation (Boel *et al*., 2019; Dewachter *et al*., 2021).

### Streamlined cell‐free syntheses

In parallel with these advances, we are now in a new age of cell‐free biology, allowing applications of three variations on the theme: Not only authentic cell‐free transcription–translation systems, as just discussed but also protein‐based and nucleic acids‐based systems (Noireaux and Liu, 2020). Protein‐based streamlined cell‐free systems can be used to produce fine chemicals as well as precursors of heavy industry‐relevant compounds. As these processes do not require the industrial setup to sustain life, they tolerate the presence of otherwise toxic molecules (Rasor *et al*., 2021). Among these, a very innovative approach has been explored early on. Rather than reconstituting *in vitro* the conditions prevailing *in vivo*, the idea, intended to improve industrial metabolic engineering, was to find a way of isolating all the enzymes needed to put together a metabolic pathway in a common container. Interestingly, the container could be of considerable size, an industrial reactor, for example. It is obviously time‐consuming to synthesize and purify individually all the enzymes of a pathway of interest. Why not use the established outcome of natural selection, with all enzymes in correct proportions in relevant cells, while getting rid of all components not required in the pathway? The operation would develop into two steps: (i) growing cells at a high density under conditions when the pathway is fully functional and boosted for production of the metabolites of interest; and (ii) making a whole extract of the cells, finding some intelligent ways to separate the metabolic fraction from the bulk of the cell's machinery, and then finding a way to keep in a reactor only those enzymes that are required in the pathway.

This can be achieved after ‘parasitic’ reactions have been identified if one can discard the parasitic elements. Panke and co‐workers proposed an astute approach to solve this quandary. In a first analysis, they boosted, *via* standard SynBio approaches, the pathway of interest. So doing, they also monitored all the changes that went up in parallel, drawing key resources away from the required pathway (Bujara and Panke, 2012). This interference was mediated by proteins, mainly enzymes and their regulators. How to get rid of them? Inactivating the corresponding genes directly would jeopardize production *via* destroying the network of interactions that make the cell functional. However, it is possible to tag the corresponding proteins by including in their genes, at positions that do not interfere with function (Oesterle *et al*., 2017), sites that code for protease‐sensitive peptide motifs. In this way, when the cell extract is obtained, it can be incubated with a protease acting specifically on the tagged regions and, thus, removing unwanted proteins. After a purification step, the extract essentially contains the enzymes of the pathway of interest and, with adequate stabilization, can be used in a chemical reactor.

In line with this approach, another family of advances is being developed. When extracted directly from living cells, cell‐free systems always develop endogenous metabolic activity. The details of this activity are not yet understood but, as just de**v**eloped, they interfere with the production of the compounds of interest. Using metabolomics to characterize time‐dependent metabolic changes in cell‐free systems and their components, including significant metabolic activity in central carbon and amino acid metabolism, Styczynski and colleagues developed several approaches to prepare extracts prior to production development. They observed that while changing the starting state of the reaction via pre‐incubation of the lysate has an impact on protein production, its impact on the metabolic state was comparatively small. Changes in lysate preparation had a greater effect on protein yield and time‐dependent metabolic profiles, while general metabolic trends were maintained. Finally, while targeted supplementation of metabolic enzymes improved protein production, endogenous metabolic activity remained resistant to these enzymatic perturbations, calling for further development to improve the processes of interest (Miguez *et al*., 2021). We can expect that in the next decade or so these families of approaches, combined in a variety of sequential reactors, including some involved in standard chemistry, will create novel designs for fine chemical syntheses.

### Evolution *in vitro*

Another crucial family of experiments in cell‐free systems is the exploration of evolution, a feature recognized as critical to biology and commonly exploited *in vivo*. It is indeed possible to develop evolution experiments in cell‐free conditions. As an illustration, Spiegelman again took an aliquot of the Qβ RNA synthesized in the test tube and added it to a second identical text tube, then repeated the procedure 15 times. At the end of this sequence, the original RNA was almost completely diluted out by the transfers, leaving in the final tube RNA that was all generated *in vitro*. Repeating these experiments allowed him to witness the evolution of RNAs. By varying the selective pressure on the system (such as temperature, nutrient mix or time allowed for reproduction), he could generate mutant RNA molecules with a wide range of properties. One of those, for instance, gradually got trimmed down from the original 3300 nucleotides to 470 – just sufficient to bind the replicase and replicate the RNA without further loss of its sequence. This small molecule had shed sections that coded for the virus' protein coat and other components it did not need in the artificial environment (Levisohn and Spiegelman, 1969). It should be noted that processes similar to this cell‐free evolution also occur *in vivo* and are important for recombination, for example, with existing viruses such as SARS‐CoV‐2. This largely explains the ability of viruses to evade innate and acquired immunity, as observed several decades ago (Meyer and Southern, 1997). In parallel with functional copies of the authentic virus, cells keep shedding short counterparts that essentially kept the 3' and 5' end required to initiate and terminate replication (Gribble *et al*., 2021). This indicates that the process of viral replication is prone to produce a large number of variants that could be exploited for use in cell‐free expression systems. Based on this early work and recent observations, advances in the *in vitro* synthesis and evolution of DNA, RNA and polypeptides have already been designed to accelerate the construction of biopolymers, pathways and organisms with new functions (Forster and Church, 2007). This trend is set to continue.

A most important sequel of Spiegelman’s early experiment has been the systematic evolution of ligands by exponential enrichment (SELEX) procedure devised by Larry Gold and his colleagues (Tuerk *et al*., 1992). After the discovery of ribozymes, it was clear that RNA molecules could fold into complex structures able to bind foreign molecules [aptamers, riboswitches and ribozymes (Ge and Marchisio, 2021)]. This prompted exploration of novel RNA‐based activities, substrate‐dependent ribozymes in particular. Using a variety of *ad hoc* selection procedures, SELEX allowed isolating functional RNAs from a pool of random sequences of RNA, in particular endowed of catalytic activities. Among the critical functions, as discussed above, the mimicry of aminoacyl‐tRNA synthetases by ribozymes was an interesting target. Perhaps the first example of ribozymes capable of charging certain amino acids onto RNA was developed by Yarus and co‐workers. They isolated synthetic ribozymes that catalyse aminoacylation of their own CCG‐terminal 2’(3’)‐OH with phenylalanyl‐AMP or tyrosyl‐AMP as an aminoacylation donor (Illangasekare *et al*., 1995). However, this early class of ribozymes was still unable to aminoacylate the CCA 3’ end of a tRNA. Using a first family of ribozymes, Suga and co‐workers designed variants of an artificial ribozyme able to bind CCA motifs and comprising random segments. These RNA molecules were submitted to a selection procedure meant to isolate those which got the ability to aminoacylate various tRNAs with phenylalanine or non‐natural derivatives (Fig. 2). One of those was able to aminoacylate its cognate tRNA with a high degree of specificity, while failing to aminoacylate non‐cognate tRNAs. Further evolution *in vitro* allowed the authors to load analogues of phenylalanine of a specific tRNA. Changing the tRNA load specificity could be easily programmed into such flexizymes, fashioning them into custom‐made catalysts to generate non‐natural aminoacyl‐tRNAs (Ramaswamy *et al*., 2004). This family of advances, based on *in vitro* RNA evolution, is likely to see considerable developments in the near future (Kofman *et al*., 2021).

All these advances require complex laboratory facilities, with development time limited by the constraints of physical experiments, hours, days, months or even years. As with a considerable number of existing technological developments, it is now possible to replace approaches that involve wet laboratories with computer‐based exploration. State‐of‐the‐art computer modelling and design technologies allow us to achieve results that engineers could not even dream of a couple of decades ago. For example, the manufacture of environmentally interesting devices starts with the succession, concept, model, computer‐aided testing of functions and resilience, and finally the creation of a prototype, with feedback to material experiments. The conceptual development of an aircraft starts from a creative search stage: choice of style, composite approach, creation of sketches and drawing of the future model, and all this can be done with the help of computers (Abbasov, 2019). While laboratory experiments were the rule rather than the exception, it soon became apparent that, starting with data analysis and storage, true *in silico* experiments – using computers – could and should be developed.

## Synthetic biology *in silico*

*In silico* biology is now a major domain of SynBio developments, with one caveat. The exponential development of computers and the speed of related advances relative to the time required to obtain results from laboratory experiments have led to the creation of a large community of researchers and engineers who claim to be able to substitute *in silico* biology for experimental (‘wet’) biology. Alas, this is often at the cost of understanding what life is. The main consequence of the shift from *in vivo*/*in vitro* to *in silico* research is that a significant fraction of the community has turned inward, with its own journals and evaluation rules, reviewing and evaluating the work it produces without the critical steps that would require predictions or explanations – validation – based on authentic biological experiments. Furthermore, the very fact that *in silico* biology – modelling in particular – requires skills in both mathematics/computer sciences and biology means that a great many studies escape proper evaluation (when they are about biology, they are considered to be computer sciences, and vice versa). This results in a large amount of work which is not relevant for answering the questions posed by SynBio (Danchin *et al*., 2018). This downside has a considerable impact when we have to follow the explosion of articles in the domain. It is virtually impossible to go beyond scraping the surface of the amount of literature claiming to be *in silico* biology. At the risk of missing important work, we have tried here to avoid this pitfall by giving an overview of a limited number of the work and trends that may provide constructive developments in the near future.

Mathematics/computer‐based models of a variety of processes essential to life have long been developed, with two different aims. For diagnostic purposes, models were meant to produce a phenomenological account of the dynamics of processes of interest. The purpose of such *simulations* was not to understand their causes but to allow researchers to make predictions about the short‐term and sometimes long‐term outcome of the processes. This family of approaches is well established. It is still witnessing substantial developments, in particular in the domain of health‐related studies.

### Simulation vs. explanation

In the context of SynBio, simulations can be used as a first pass meant to measure whether a particular family of experiments behaves consistently as predicted, thus substantiating the relevance of the collected data and pointing out missing elements. The power of this family of models parallels the continuous advances of computing capacities associated with the continuous emergence of faster computers. In recent times, artificial intelligence (AI) approaches based on deep learning flourished as tools of choice in the domain of diagnosis (Akay and Hess, 2019). Many AI‐based models are currently limited to diagnosis because successful predictions are not sufficient to tell correlation from causality, in particular when the underlying reasons for a prediction ‘success’ cannot be traced easily. To make the most of these models and use them as help to discovery requires that the outcome of their operation can be traced back to a causality chain. This restriction explains why legal regulatory instances have now asked creators of AI‐based models to be able to highlight the internal chain of causality in their successful models. This is understood as a way to associate prediction with understanding [https://eur‐lex.europa.eu/legal‐content/EN/TXT/?uri=CELEX:52021PC0206 and for an example of the way understanding can be visualized in an AI model, see Prifti *et al*. (2020)]. This limitation should be extended to a vast number of models. Here is an illustration of the difference between phenomenology (simulations) and identification of causality (rational scientific modelling).

The behaviour of cells on plates is often used to illustrate SynBio construction [see a famous early illustration in (Elowitz and Leibler, 2000)]. However, a large number of phenomenological models can be used to account for this behaviour (McCallum and Potvin‐Trottier, 2021). For example, it has long been observed that colonies on plates often generate patterns that have dendritic shapes (Fall *et al*., 2004). This is generally ‘explained’ by models involving repulsion, multi‐scale patterns, self‐organization and all kinds of buzzwords [see, e.g. Deng *et al*. (2014)]. Yet, analysis of the causal chain of events that lead to these patterns demonstrates that the explanation of the apparent repulsion is not mediated by any ‘self‐organization’ magic force. Notwithstanding a possible Marangoni effect, if movement involves surfactants and quorum sensing (Daniels *et al*., 2006), ‘repulsion’ is driven by the – almost trivial – role of the nutrient source in the cell’s behaviour: Bacteria need nutrients to multiply. They evolved a chemotactic response that makes them move towards nutrient sources (and, therefore, avoid regions where they have been depleted). Understanding the authentic chain of causality is important: What appears to the naïve mind as a self‐organized repulsion is in fact the distribution of moving cells in regions where nutrients are available (Sekowska *et al*., 2009). This experiment illustrates the dichotomy of modelling, which can lead either to phenomenology or to understanding; the latter being the only way to generate real advances. To move beyond the phenomenology of simulations, one must interpret the data critically, looking for genuine causality and refraining from appealing to folk magic.

### Standardization

To make the most of computer‐mediated exploration, it is critical that models are built on very precise definitions and cardinal foundations, not on fuzzy vocabulary. This topic, in particular, the need of accurate definitions, is discussed in details by Clark and Hicks in the field of chemistry, showing how the choice of models impacts the outcomes of research, including with modern AI approaches (Clark and Hicks, 2020). Because models rest on the collection of relevant data, the key question of standardization and nomenclature must be asked, with expedient approaches (Decoene *et al*., 2018). A great many developments of SynBio are based on manipulation of nucleic acids. Because these polymers are made of the chaining of elementary building block, they are generally handled by computers as are abstract linear alphabetic texts. Intelligent storage and analysis of nucleic acid and also protein sequence data began four decades ago (Blumenthal *et al*., 1982). Subsequently, an explosion of applications resulted in the creation of a new domain of biology, bioinformatics, with a slight change in the initial meaning of the word, now often restricted to genome‐based studies (Hogeweg, 2011). Yet, while this restriction is commonplace, the advent of genomics, with its related ‘omics’ studies (Yadav, 2007), has considerably expanded the domain, with only fuzzy boundaries with recently fashionable domains such as AI and image analysis (Burian *et al*., 2021).

Gene, genome and some transcriptomics data are collected, managed and released in a highly standardized way by the International Nucleotide Sequence Database Collaboration (DNA Database of Japan, Mishima, Japan; European Nucleotide Archive, Hinxton, UK; and GenBank, Bethesda, USA), which has developed standards for more than three decades (Arita *et al*., 2021), and controlled vocabulary is implemented for proteins by UniProtKB (Feuermann *et al*., 2021). The Gene Ontology Consortium maintains a knowledge base that ‘*develops a comprehensive, computational model of biological systems, ranging from the molecular to the organism level, across the multiplicity of species in the tree of life*’, also based on controlled vocabulary (Gene Ontology Consortium, 2021). However, while the nomenclature of chemicals and chemical reactions tend to follow well‐accepted rules [such as in CheBi (Hastings *et al*., 2016) or Rhea (Lombardot *et al*., 2019) for example], with efforts to allow interoperability (Navelkar *et al*., 2021), the nomenclature of enzymes – besides the EC number standard – is still quite variable. Gene names are considerably dependent on the goodwill of authors and this has considerable negative impact on sequence annotation (and, therefore, discovery). Also, the data structure of specialized databases is still extremely disparate, which prevents interoperability (Danchin *et al*., 2018). When dealing with SynBio, the situation is possibly less disappointing. The iGEM repository of Biobricks (https://biobricks.org) and, in Europe, the SEVA effort (Martínez‐García *et al*., 2020) are pushing hard for widespread standardization. A further incentive for an international coordinated effort in this direction would be more than welcome to support the development of SynBio in the next decades.

All the same, three major domains of *in silico* research for SynBio develop in parallel: ‘omics‐based’ SynBio, whole‐cell models and *in silico* integration of metabolic pathways.

### ‘Omics‐based’ synthetic biology

Besides the domain of genomics, born and diversifying into a number of subdomains, the fields of transcriptomics, proteomics and metabolomics that progressively sprouted out of this original domain (Tyo *et al*., 2010; Pühler, 2012) are now key for SynBio developments. The high‐dimensional datasets generated by these experimental approaches require considerable storage capacity and widespread sharing. A large number of analysis methods must also be made available. Sometimes it is possible to combine both storage and availability of analysis methods. In the case of bacterial genome data, this is the case, for example, with the MicroScope platform and its implementation of the MaGe methods (Vallenet *et al*., 2020). Repositories such as GitHub with its community of open‐source developers are progressively gaining more and more impact (see, e.g. its SynBio entry: https://github.com/topics/synthetic‐biology). Cloud computing provides generally low‐cost and highly flexible solutions in the domain of molecular modelling, omics data analytics (e.g. RNA sequencing, metabolomics or proteomics datasets) and for the integration, analysis and interpretation of phenomes (Koppad *et al*., 2021). Merging highly heterogeneous datasets has been a prime goal early on (Arakawa and Tomita, 2013). This is critical because multi‐omics may be used to improve systematic genome manipulation for novel synthetic constructs (Fontana *et al*., 2020) or improve industrial processes. Among the many illustrations of the approaches for SynBio applications, we may notice fine cellulose biosynthesis (Ryngajłło *et al*., 2020), treatment of natural biomass, in particular lignocellulose (Xie *et al*., 2014; Chen and Dou, 2016), or in the present context of general concern triggered by climate change, genome manipulation carried out for the metabolic engineering of Cyanobacteria (Hagemann and Hess, 2018; Ng *et al*., 2020).

The data introduced in modern omics analyses allow for efficient characterization of the genetic, regulatory and metabolic phenotypes of engineered microbes. Yet, designing genetic interventions to achieve the desired phenotype remains a challenge. With recent developments in genetic engineering techniques, the time frames associated with constructing and testing strain designs have been greatly reduced through *in silico* modelling. This has created an efficient design–build–test–learn loop iteration cycle between experiment and analysis. However, the scale and complexity associated with multi‐omics datasets still require manual biological reasoning about the mechanisms driving phenotypic changes. As a consequence, the use of traditional statistical approaches can still reduce the dimensionality of these datasets and help compare strains of interest and improve metabolic engineering (Danchin *et al*., 2018; St John and Bomble, 2019).

Besides modelling individual cells, modelling is also used to understand how microbial communities and their synthetic counterparts may form stable ensembles. Models allow researchers to better monitor the changes in microbiome composition and, for example, how this affects disease onset and development. Construction of models that integrate genomics, transcriptomics, proteomics and metabolomics allows researchers to visualize the meta‐metabolism of the community. Constraint programming (Brown and Miguel, 2006) is used to couple meta‐metabolism and multi‐omics analyses (Ebrahim *et al*., 2013). As an illustration, COBRA modelling combined with meta‐omics analyses and multivariate statistical analyses are used as a tool to add value to clinical trials and ultimately propose therapeutic interventions (Heinken *et al*., 2021). This type of work, when submitted to relevant constraints, allowed construction of a synthetic gut microbiome (Mabwi *et al*., 2021).

Finally, we have seen that miniaturization was involved in developing novel approaches for parallel evolution of microbial cultures. This domain is also used to explore the consequences of the use of novel technologies based on new materials, for example, nanomaterials, which, because of their size, may be a matter of concern. Modelling using multi‐omics approaches explored the impact of nanomaterials on cells or pathways *via* interpretation of omics data (Mortimer *et al*., 2021).

### Simulating whole‐cell behaviour

In parallel with the sequencing of whole genomes, researchers proposed to shift efforts from *in vivo* biology to reconstitution of whole cell in computer‐mediated models. Early attempts focused on *E. coli* at a time when DNA sequencing was not yet available (Weinberg and Berkus, 1971). The revolution brought about by sequencing resulted in a nucleic acid‐, then genome‐centric view of cells, but models were initially restricted to simulations based on the way macromolecule and intermediary metabolism could integrate functions previously identified from the study of individual genes.

As an early illustration of the integrated behaviour of cells, modelling was developed around a generic software environment that would explore, from the knowledge of their genome, how cells behave in all their biological properties. A first simulation software pipeline, E‐Cell, was made publicly available in 1997, poised to exploit the then recently acquired genome data of *Mycoplasma genitalium* (Tomita *et al*., 1997). This generic software environment for simulating a cell was subsequently improved to conduct *in silico* experiments based on numerical data obtained from *in vivo* experiments involving other organisms (Tomita *et al*., 1997; Tomita, 2001; Ishii *et al*., 2004). Among applications of the software was the exploration of an osmoregulatory switch in the growth properties of *E. coli* (Srividhya and Krishnaswamy, 2004) and the deciphering of red blood cells’ metabolism (Yachie‐Kinoshita *et al*., 2010). The domain of mathematics involved in computation was based on differential equations. This preference is not without consequences, as it implies that biological objects are assumed to display a continuous behaviour, with their concentration represented as a real number not as a discontinuous integer. While this is good enough for objects having a concentration in the millimolar range or above, this is inappropriate when individual objects must be identified, such as regulatory molecules. For example, regulators or co‐factors have often a concentration in the sub‐micromolar range, or are present in cells such as an individual *E. coli* in less than a handful of individual molecules. Ten years after its start, the E‐Cell project experienced a thorough reorganization, now focusing on simulations meant to help industrial applications. This focus on simulation rather than understanding may explain why, after a series of improvements, the project, while still existing (https://www.e‐cell.org/about.html) progressively stalled. At present, the whole‐cell modelling of *E. coli* follows another turn, still based on standard approaches but with some input of stochasticity to take into account entities that are present at a low number in cells (Sun *et al*., 2021).

More fitting to SynBio approaches, whole‐cell models may be used as a basis for genome design based on previous knowledge. The bacterium *Mycoplasma genitalium* is represented by an updated whole‐cell model (https://github.com/CovertLab/WholeCell). At this time, this framework combines 28 cellular submodels, integrating all the known functions of every gene and molecule in the cell, running individual models on multiple supercomputers (Rees‐Garbutt *et al*., 2021). This design allows researchers to propose knockouts of individual protein‐coding gene, phasing out genes previously identified by their importance for growth and to test possible phenotypes to identify synthetic lethal associations. This semi‐empiric work depends on known phenotypes, conducting whole‐cell model simulations in three steps: design (the outputs of algorithms select possible gene deletions), simulation (behaviour of the genome minus those deletions) and testing (the behaviour of the resulting cell is analysed). Several simulations are conducted in parallel. Those that removed the most genes from the genome and produced cells that were still able to divide are kept for further study. Once this step is completed, the software proceeds to the next cycle, increasing the number of gene deletions and generating progressively smaller genomes which can be subsequently used as synthetic constructs. This bypasses the development of slower experimental approaches based on the construction of multiple knockout mutants, such as those which uncovered hidden reactions in *E. coli* (Nakahigashi *et al*., 2009), showing how enzyme promiscuity could combine with paralogous metabolism (Chan *et al*., 2014; Guzmán *et al*., 2019) to generate unexpected pathways. Similar genome‐scale modelling is also developed using Eukarya such as Yarrowia (Czajka *et al*., 2021).

Based on genome data, these models are very general but not realistic. Their properties of symmetry do not take into account the asymmetry of the cell’s geometry. Cells are generally not spheres, and taking into account the real time‐dependent distribution of their metabolites is far from straightforward. Furthermore, compartmentalization is a critical feature of the development of metabolism (de Lorenzo *et al*., 2015; Bohrer and Xiao, 2020) and this implies that realistic models must make the most of representations that consider the cells’ shape and the way they divide. Exactly as illustrated previously with the case of populations of cells moving on plates, many phenomenological models try to fit with 3D observations to represent the cell’s geometry. Bacteria are often rod‐like structures. Analysis of the constraints that account for this family of shapes provides interesting views [see, e.g. Chang and Huang (2014); Banerjee *et al*. (2016)]. Yet, the ultimately disparate causes resulting in bacteria being rods have a common source based on metabolism: Growth must put together compartments that have widely different spatial properties, while the core of metabolism that generates major building blocks is likely to grow in three dimensions. Indeed, growth of the cytoplasm is three dimensional, while membranes are two‐dimensional, and the genome is unidimensional. The major source of building blocks is expressed in the cytoplasm, and therefore, grows in three dimensions, unless specifically regulated or organized metabolic limitations prevent non‐homothetic growth of the various cell’s compartments. Remarkably, it is cytosine metabolism (and more generally *de novo* pyrimidine synthesis) that overcomes this obstacle, with all pyrimidine‐related pathways converging on the activity of CTP synthetase (Ou *et al*., 2020; Danchin, 2021). The consequences of this metabolic adaptation have not yet been input in models of bacteria *in silico*, but this will have considerable impact for the future of SynBio metabolic engineering developments. Many microbes have a structure that departs from that of spheres or bacillus shapes. They make filaments during their growth, either under natural circumstances (e.g. streptomycetes and filamentous fungi), but they also make filaments under stressful conditions or during ageing. This phenomenon is important for cell factories, and phenomenological models have been proposed to understand and control filamentation, with the aim of designing the morphogenesis of fungi (Meyer *et al*., 2021) or Cyanobacteria (Yamamoto *et al*., 2021). The process of filamentation has been studied for a long time, but the link to metabolism has generally not been explored, apart from the identification of targets such as LexA in bacteria, with limited *in silico* modelling (Bellio *et al*., 2020).

Finally, in yet another register, modelling was used to improve cell‐free protein synthesis, in a typical application of *in silico* approaches to complement *in vitro* experiments (Müller *et al*., 2020). For example, the domain of heterologous protein expression benefited from approaches allowing codon optimization, a domain explored at the very beginning of genomics (Ang *et al*., 2016). At a finer level of granularity, the evolution of proteins was modelled as a function of their abundance as well as environmental parameters such as temperature. Interestingly, in addition to their expression level, protein evolution is determined by the concentration of chaperones, highlighting the critical role of these neglected essential factors (Agozzino and Dill, 2018). More in line with larger scale physiological data, integration of a variety of approaches investigated the role of stress and helped in the improvement of recombinant protein synthesis (Chen *et al*., 2021c). At this point, we may understand that we are still a long way from having whole‐cell models that go far beyond simulation. Nevertheless, modelling can be successfully used to guide SynBio designs. Moreover, based on semi‐empirical approaches, intermediary metabolism is now more suitable for building predictive models that help streamline existing metabolism while exploring the novel avenues we mentioned earlier.

### Whole‐cell metabolism

It is common to assume that metabolism follows textbook pathways. Yet, unexpected metabolic pathways are the rule rather than the exception and the metabolic capacity of virtually all organisms is vastly underappreciated (Medema *et al*., 2021). How can we connect our knowledge of genomes and whole‐cell metabolism? Back in the XIX^th^ century, August Weisman postulated that living organisms combined two major characters, with a *germen*, that was responsible for the hereditary properties of the organism and a *soma* that harboured the germen while displaying observable features. This distinction is still valid and recognized as the genotype/phenotype dichotomy. With the advent of DNA sequencing and identification of the genome sequence, this dichotomy is now often referred to as the genome/phenome question, and this is the subject of explicit *in silico* investigations (Norsigian *et al*., 2020). Metabolic engineering, developed along an integrated view of the genome/phenome dichotomy, associates traditional metabolic engineering (Bailey, 1991) with systems biology, synthetic biology and evolutionary engineering (Jang *et al*., 2019). It is enabling the development of microbial cell factories capable of producing efficiently a myriad of chemicals and materials, including biofuels, bulk and fine chemicals, polymers, amino acids, natural products and drugs. These developments are based on integrated circuits within metabolic networks, preferably using multi‐omics high‐throughput data collection (Chen and Li, 2016).

Combined with genome engineering and genome‐wide metabolic simulations, as discussed above, many tools and strategies have been developed to generate microbial cell factories (Ko *et al*., 2020). Here, we focus on a family of models that can be used as predictive tools to assess metabolic constructs. Based on flux balance analysis [FBA, (Edwards and Palsson, 1998)], genome‐scale metabolic models [GSMM (Edwards and Palsson, 1999; Xu *et al*., 2013)] have been developed to explore the metabolism of a large number of microorganisms of industrial interest, such as *P. putida* (Belda *et al*., 2016) or *B. subtilis* (Belda *et al*., 2013). Being based on the general constraints of stoichiometry (Moyer *et al*., 2021), FBA is generic and may accommodate the network structure of any metabolic pathway (Antoniewicz, 2021). For example, the details of the complex metabolism of actinomycetes are now clarified, allowing metabolic engineering in these organisms (Palazzotto *et al*., 2019). Furthermore, these applications are not restricted to bacteria: Flux balance analysis guided the evolution of a yeast chassis (Pereira *et al*., 2021).

Other genome‐wide metabolism models have been implemented to explore *in silico* the metabolism of short‐genome bacteria such as *Mesoplasma florum*. Using sequence and structure homology, the set of metabolic functions that its genome encodes was identified, allowing the reconstruction of a metabolic network representing one third of its protein‐coding genes. Simplification of the growth medium allowed quantification of substrate uptake and product secretion rates, which, together with the experimental biomass composition, were integrated as species‐specific constraints to produce a functional model of metabolism at the genome scale (Lachance *et al*., 2021). At a larger scale, a whole‐cell modelling with emphasis on metabolism is continuously improved for the model *E. coli* (Sun *et al*., 2021). This effort is particularly important because *E. coli* remains the best known organism, with continued progress in understanding the functions encoded in its genome, based on laboratory experiments.

Modelling of metabolism is also useful to explore communities of organisms (Vázquez‐Castellanos *et al*., 2019). This family of approaches is particularly timely because we are beginning to understand, through the study of seawater, what metabolic ‘currencies’ are shared by the millions of microbes that coexist in this environment (Durham, 2021). Microbial co‐cultures have been studied using FBA, with interesting observations involving cross‐feeding (Konstantinidis *et al*., 2021). Another type of interaction between microbes is witnessed by the role of viruses, which are able to reprogram the metabolism of their host (Jacobson *et al*., 2021). Understanding the corresponding processes may allow construction of specific pathways for metabolic engineering.

### Evolution *in silico*

Many evolutionary experiments use model organisms, but they are slow (Sekowska *et al*., 2016; Grant *et al*., 2021) and the study of evolution *in silico* remains the approach of choice. Adaptive evolution in the laboratory has been developed for organisms of industrial interest. The process benefits greatly from *in silico* modelling (Lee and Kim, 2020). In this context, exploration of the potential deleterious effects of synthetic mobile elements in genomes has been studied *in silico* (Zamdborg *et al*., 2015) and early work was designed to predict the evolution of the virulence of bacteria of interest to agriculture (Strauß *et al*., 2016). In line with the objectives of SynBio, based on families of enzyme variants, modelling is used to design synthetic counterparts with *ad hoc* catalytic properties (Broom *et al*., 2020; Bunzel *et al*., 2021). More generally, *in silico* modelling allows engineers to explore the evolutionary landscape along lines that would be impossible to follow in wet lab experiments, particularly when several alternative pathways are possible (Ambrus *et al*., 2020). Furthermore, frequent evolution by gene duplication creates unexpected avenues that must be explored for the consequences of the regulatory properties they create (Marchant *et al*., 2019).

Understanding gene networks is crucial for interpreting evolution and predicting evolutionary pathways (Gautam and Kumar Sinha, 2021). This task is difficult in the presence of noise, a pervasive feature, and requires special treatment (Vatsa and Agarwal, 2021). It is particularly important to understand the evolution of regulator binding sites. Comparative analysis of bacterial genomes has revealed that such sites are rapidly created at random during evolution, and then retained when they have a positive effect on the organism. Interestingly, the patterns of evolution depend on the nature of the regulator (Mrázek and Karls, 2019). A parallel software package, GeNESiS, was designed for the modelling and simulation of the evolution of gene regulatory networks (Kratz *et al*., 2008). Various models of gene regulatory networks are available (Santibáñez *et al*., 2020; Chen *et al*., 2021b), in particular dynamic models will be of interest to take evolution into account (Handzlik *et al*., 2021). Analyses involving co‐evolution patterns *via* comparative genomics (Zhang *et al*., 2021a; Guo and Amir, 2021) will considerably benefit SynBio studies.

## Conclusions

The development of SynBio is at a turning point. Over the past two decades or so, a large number of proofs of concept have been established, demonstrating the feasibility of a considerable number of SynBio developments. We are now at a point where even the most far‐fetched ideas can be implemented in constructions of considerable industrial interest. The integration of approaches combining experimentation with living chassis, cell‐free synthesis and modelling has created a virtuous cycle of design, construction, testing and learning loops between experiments and analysis, combining physiology, genetics, biochemistry and bioinformatics in a way that is constantly progressing. Perhaps the most promising approaches in terms of industrial production are experiments using building blocks that differ from those established during the evolution of life. It also seems important to open up strategies that combine biology and chemistry, for example, in factories where a succession of reactors combining *in vivo* fermenters and biochemical and chemical reactors creates a production line starting with low‐cost inputs and leading to high‐value products. At the same time, understanding the processes will greatly improve the safety of the facilities, with prevention locks in place that will foil the unwanted spread of chemical or biological contamination. In particular, it is essential to remember that the closer we get to existing living organisms, the more difficult it is to control their spread. Contrary to popular belief, artifice is much less dangerous than nature (Danchin, 2016).

## Funding information

No funding information provided.

## Conflict of interest

None declared.
